# Plant Hormesis Management with Biostimulants of Biotic Origin in Agriculture

**DOI:** 10.3389/fpls.2017.01762

**Published:** 2017-10-13

**Authors:** Marcela Vargas-Hernandez, Israel Macias-Bobadilla, Ramon G. Guevara-Gonzalez, Sergio de J. Romero-Gomez, Enrique Rico-Garcia, Rosalia V. Ocampo-Velazquez, Luz de L. Alvarez-Arquieta, Irineo Torres-Pacheco

**Affiliations:** ^1^Laboratory of Biosystems Engineering, Autonomous University of Queretaro, Faculty of Engineering, Campus Amazcala, Queretaro, Mexico; ^2^Laboratory of Microbiology, Autonomous University of Queretaro, Faculty of Chemistry, C.U. Cerro de las Campanas, Queretaro, Mexico

**Keywords:** hormesis, agriculture, nutraceutic, elicitor, homolog DNA

## Abstract

Over time plants developed complex mechanisms in order to adapt themselves to the environment. Plant innate immunity is one of the most important mechanisms for the environmental adaptation. A myriad of secondary metabolites with nutraceutical features are produced by the plant immune system in order to get adaptation to new environments that provoke stress (stressors). Hormesis is a phenomenon by which a stressor (i.e., toxins, herbicides, etc.) stimulates the cellular stress response, including secondary metabolites production, in order to help organisms to establish adaptive responses. Hormetins of biotic origin (i.e., biostimulants or biological control compounds), in certain doses might enhance plant performance, however, in excessive doses they are commonly deleterious. Biostimulants or biological control compounds of biotic origin are called “elicitors” that have widely been studied as inducers of plant tolerance to biotic and abiotic stresses. The plant response toward elicitors is reminiscent of hormetic responses toward toxins in several organisms. Thus, controlled management of hormetic responses in plants using these types of compounds is expected to be an important tool to increase nutraceutical quality of plant food and trying to minimize negative effects on yields. The aim of this review is to analyze the potential for agriculture that the use of biostimulants and biological control compounds of biotic origin could have in the management of the plant hormesis. The use of homolog DNA as biostimulant or biological control compound in crop production is also discussed.

## Introduction

Currently, there are generalizable processes from which different terminologies have been constructed, including those from which they may be described; hormesis is a process of this type, present in all organisms (Calabrese et al., 2007). In toxicology, hormesis is defined as a biphasic response to a toxic compound (stressor), which at low doses induces a beneficial effect and at high doses produces a toxic effect. However, at the physiological level, this can be translated as an adaptive response of an organism to a low level of stress factor, accompanied by overcompensation, when the homeostasis readjustment has been interrupted (Calabrese et al., 2007; Mattson, 2008; Calabrese, 2009). This allows the organism to acclimate to its new environment, a key factor in the evolutionary process. The factors responsible for inducing hormesis are known as hormetins or stressors. In this sense, it has been established that in plants the challenge with different levels of stress constitutes an adaptive process, having reminiscence with the phenomenon of hormesis abovementioned. This stress can be established as “eustress” (beneficial stress) if the effect is similar to the hormetic effect in low doses of a toxin, or “distress” (harmful stress) if the level of this generates an irreversible or negative damage in the plant (Hideg et al., 2013). The level of eustress or distress toward the same factor (e.g., a biostimulant) is not always the same due to the process of adaptation of the plants, thus it is important to take into account these terms when talking about hormesis to establish a strict difference between low dose and high dose of a hormetic factor. It is considered potentially toxic an agent that disrupts homeostasis, and the hormetic effect can be observed as a reparative process that slightly or modestly overshoots the original homeostatic level (Calabrese et al., 2007). In this sense, several plant stressor of biotic origin disrupt homeostasis at molecular level by inducing adaptive responses in organisms that cause increased growth and induce defense processes against biotic and abiotic stresses in several crops, although at cellular level the effects might not clearly been observed by some elicitors. Great efforts have been made to define the concept of biostimulant. According to du Jardin (2015), those compounds or microorganisms that have the function of improving nutrition, efficiency, and tolerance to abiotic stress and/or quality traits of crops are called biostimulants. A more recent definition is that proposed by Yakhin et al. (2017): “A formulated product of biological origin that improves plant productivity as a consequence of the novel, or emergent properties of the complex of constituents, and not as a sole consequence of the presence of known essential plant nutrients, plant growth regulators, or plant protective compounds.” Some of the key elements of its definition are the composition of biotic origin, its ability to modify physiological processes to increase the productivity of plants, and protect them from abiotic stress (Yakhin et al., 2017). However, compounds protecting plants from biotic stresses are called biological control compounds. It should be clarified that many of the biotic origin compounds not only have the ability to protect plants from either abiotic or biotic stress, but against both, it is to say they have a function of both biostimulant and biological control compounds (Lucas et al., 2014). If the biostimulant or biological control compound is of biotic origin it is called elicitor (du Jardin, 2015). Elicitors are factors that trigger plant immunity in a dose–response manner. Low dose of elicitors normally induces a eustress condition and at high dose a distress in plants (Mandal et al., 2013; Garcia-Mier et al., 2015; Zunun-Pérez et al., 2017). This behavior is similar to the hormetic effect of physical and chemical factors (Hooper et al., 2010; Tierranegra-García et al., 2011; Mejía-Teniente et al., 2013; Baenas et al., 2014; Calabrese, 2014a; Liu et al., 2016). In order to unify concepts in this sense, it would be necessary to carry out experimentation evaluating elicitor’s dose–response curves to determine the hormetic effects of these compounds. Thus, it is clear that mild-stimuli activates plant defense provoking a eustress situation using an elicitor. The stresses coped by plants may have a biotic or abiotic origin provoking an increase in metabolites to cope the stress (Tierranegra-García et al., 2011; Mejía-Teniente et al., 2013; Baenas et al., 2014; Liu et al., 2016); however, when an individual feeds on a “stressed plant” is good for health, a concept called “xenohormesis” (Hooper et al., 2010). Xenohormetic potential of crops can be increased by the hormesis management due to plant possess receptors for molecular patterns (MPs) of different origin as microbial-associated molecular patterns (MAMPs), pathogen-associated molecular patterns (PAMPs), damage-associated molecular patterns (DAMPs), and HAMPs that potentially triggers secondary metabolites pathways. The aim of this review is to analyze possibilities where eustress management using biostimulants or biological control compounds of biotic origin in plants create a hormetic condition to induce equilibrium between xenohormetic potential and yields in crops. The adequate management of this phenomenon it is considered that will be of great importance because of the climate change scenario for agriculture. The use of homolog DNA as biostimulant or biological control compound in crop production is also discussed.

## Hormetic Dose–Response In Plants

Hormesis can be defined as a biphasic response in which high doses of a toxic agent could cause inhibition while low dose of the same toxic can cause stimulation (Calabrese, 2009). This process is described by a U or J shape in which there is an initial disruption of homeostasis (i.e., toxicity) followed by a modest overcompensation response that eventually leads to a re-establishment of homeostasis. The changes suffered in cells or organisms at low doses of a stress condition reflect in environmentally induced altered phenotype, the above can be translated in a quantitatively plasticity potential (Calabrese and Mattson, 2011; Calabrese, 2014a). It is this modest overcompensation response, which is seen as the hormetic low-dose stimulation. A study carried out in *Arabidopsis thaliana* at low doses of a synthetic elicitor 2-(5-bromo-2-hydroxy-phenyl)-thiazolidine-4-carboxylic acid (BHTC) enhanced root growth, while high doses of this compound inhibited root growth, besides inducing defense (Rodriguez-Salus et al., 2016). At low levels BHTC triggers a coordinated intercompartmental transcriptional response manifested in the suppression of photosynthesis- and respiration-related genes in the nucleus, chloroplasts, and mitochondria as well as the induction of development-related nuclear genes, while at high doses induce typical defense-related transcriptional changes (Rodriguez-Salus et al., 2016). Hormesis has been widely characterized in the toxicology field, however, considerable efforts have focused on studying this process on the plant biology and agricultural areas in order to enhance crop production (Calabrese, 2014a). Also a major research need is the extension of hormesis beyond chemical stressors to abiotic (e.g., habitat) and biotic stressors (e.g., species introductions, organism interactions) (Chapman, 2001). Despite the fact that hormesis is a generalizable process that can occur in all organisms, hormesis has been primarily associated with only chemical and physical factors and a limited number of studies on plants (Calabrese and Blain, 2005; Balasubramaniyam, 2015).

The spectrum of endpoints displaying hormetic dose responses is also broad being inclusive of growth, longevity, numerous metabolic parameters, disease incidences (including cancer), and various performance endpoints such as cognitive functions, immune responses, among others (Calabrese and Blain, 2005). Reports of ca. 8000 dose responses within a hormesis database concluded that hormesis has specific characteristics, which are highly generalizable, being independent of the biological model, endpoint measurement, and chemical/physical stress inducing agent; and also the response observed typically falls within a range between approximately 30 and 60% higher than control values (Calabrese and Blain, 2005; Calabrese et al., 2007; Calabrese, 2013, 2014b). Studies in plants have been conducted mainly measuring the endpoint of growth, metabolism, mutagenic, survival, reproduction meanwhile the immune responses are less known (Calabrese and Blain, 2005). The plant hormesis have been often carried out using ion metals, herbicides, or phytotoxins (Poschenrieder et al., 2013; Belz and Duke, 2014). However, as well as the use of environment stress factors the same basic problems have been observed with the use of herbicides, the potential harmful effect in crop plants caused by the toxicity is observed (Belz and Duke, 2014). The hormesis management can be a powerful strategy to satisfy the demand of the prevailing agriculture to maintain desirable yields in crops and increase the xenohormesis potential. But it is indispensable to consider that for the assessment and characterization of the hormesis process the experimental designs require more doses, greater simple population, and a heightened need for replication (Calabrese, 2014b). The hormesis management with biostimulants or biological control compounds is founded in the recognition of plant receptors through which the induction of secondary metabolism modification can be achieved.

### Hormesis Mechanism in Plants

Generally, cellular and molecular mechanisms under the effect of hormesis include the activation of the growth factor signaling pathways, ion channels, kinases and deacetylases, and transcription factors responsible for the production of cytoprotective proteins such as chaperones [i.e., heat-shock proteins (HSPs)], antioxidant enzymes (i.e., superoxide dismutases and glutathione peroxidase), and growth factors (i.e., insulin-like growth factors and brain-derived neurotrophic factor), as well as cell survival genes (Mattson, 2008). In plants, the mechanism of hormesis is still unknown, however, some mechanisms have been proposed, (1) a stress factor may have a mode of action as a growth stimulator, (2) the mechanism of hormesis is dependent on the mechanism of the herbicide as a phytotoxin at low concentrations, and (3) a more indirect mechanism for hormesis, overcompensation, related in part with induction of plant defenses (Belz and Duke, 2014). Other authors proposed that low concentrations of toxic metals induce hormetic effects through activating plant stress defense mechanisms (Poschenrieder et al., 2013). The induction of reactive oxygen species (ROS) by mild stress (eustress) leading to the activation of antioxidant defenses, stress-signaling hormones, or adaptive growth responses is the most probable pathways for hormetic responses (Poschenrieder et al., 2013).

In plants exposure to abiotic stressors brings to oxidative stress by affecting antioxidative defense machinery, electron transport system, or induction of lipid peroxidation, however strict redox level, regulated by enzymatic and non-enzymatic antioxidants, maintains cellular redox homeostasis and control of signaling pathways (Singh et al., 2016). Biotic stressors also induce ROS production. For example, redox homeostasis is necessary for symbiosis between legumes and rhizobia, inoculation of *M. truncatula* seedlings with pathogenic or symbiotic bacteria induces the oxidative burst in the host, with a major difference in the levels of ROS production (Peleg-Grossman et al., 2012). Strawberry plant cells treated with AsES elicitor obtained from *Acremonium strictum* exhibited a triphasic production of H_2_O_2_ and a rapid intracellular accumulation of NO (Martos et al., 2015).

Some of the redox sensitive pathways, observed in organisms, which induce adaptations includes NF-κB, the MAPK family, the phosphoinositide-3-kinase (PI3K)/Akt pathway, p53 activation, and the HSPs, adaptations are also mediated by H_2_O_2_, a ROS byproduct, which upregulate gene expression (Ji, 2014). Environmental stresses like drought, heavy metals, and UV radiations enhance ROS, provoking damage in biomolecules including proteins. Production of HSPs is essential for folding and repair of damaged proteins and serves to promote cell survival conditions (Calabrese et al., 2016). HSPs respond also to biotic stresses such as pathogen infection and insect attacks (Park and Seo, 2015). Heat stress (37°C) and *Xanthomonas campestris* pv. vesicatoria infection distinctly induce CaHSP70a in pepper leaves, mediating the hypersensitive cell death response (HR) by *X. campestris* pv. vesicatoria (avrBsT) infection. Strong induction of defense- and cell death-related genes in transient CaHSP70a overexpression was also observed (Kim and Hwang, 2015). However, there is a complex and integrative array of signal transduction pathways that mediate hormetic stimulatory responses (Calabrese, 2013).

### Biostimulants and Biological Control Compounds Affecting the Hormesis Management

Hormetins according to Stebbing (1987, 1998) depends to a greater extent by organisms rather than the chemical, and then any agent that can disrupt homeostasis would be expected to induce a hormetic response to the induced damage (Calabrese, 2008). It must be also taken into account that organisms respond in a hormetic manner to signals that indicate stress, toxicity, or disruptions in homeostasis (Calabrese, 2008). Plants perceive MAMPs, DAMPs, HAMPs, or PAMPs as signals of danger and induce defense mechanisms, thus disturbing homeostasis trying to cope the potential problem.

**FIGURE 1:**
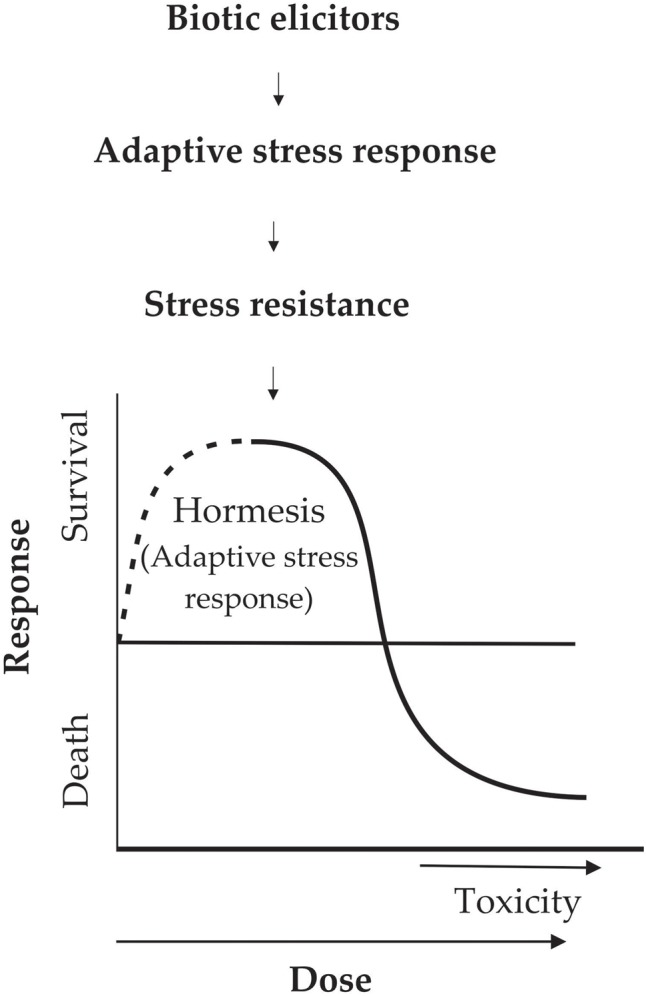
The hormesis induction through elicitors of biotic origin in plants.

Biostimulants are a proposed concept describing any substance or microorganism applied to plants with the aim to enhance nutrition efficiency, abiotic stress tolerance, and/or crop quality traits, regardless of its nutrients content (du Jardin, 2015). According to biostimulants definition among organisms either beneficial or pathogens such as bacteria, fungus, virus, nematodes, and plants may be included. It should be clarified that if we are talking about compounds that protect plants from biotic stresses they are called biological control compounds. Plants can recognize molecular of biotic origin (elicitors) because they are the signal of damage done to plants by other living organisms or environmental conditions. Little is known about the evaluation of hormesis in plants by compounds derived of organisms (Calabrese and Blain, 2005; Belz and Duke, 2014). Studies of herbicide hormesis propose that the mechanism of action is related to the target site of the herbicide, or are produced by overcompensation to moderate stress induced by the herbicides or a response to disturbed homeostasis (Belz and Duke, 2014). The hormetic dose response model in plants can lead to the determination of the biostimulant concentration in which the highest adaptive response studied is observed. The responses, which can be evaluated in hormesis, are disease resistance, production of some secondary metabolites, yields, and growth among others (Calabrese and Blain, 2005; Mattson, 2008; Calabrese and Mattson, 2011). The mechanism increasing plant fitness by biotic elicitors is inducing a defense response in plant translated in increased ROS levels leading to oxidative stress inducing disruption of the redox homeostasis and negative effects on macromolecules as proteins, DNA, RNA, and lipids, which are necessary for cell functioning (Sewelam et al., 2016). Adaptive response causes the recovery of homeostasis, by activation or repression of several defense genes and metabolic pathways (Bhattacharyya et al., 2014). Currently, there are many studies of the effect of biotic elicitors in plants. However, in these studies more doses are necessary to be evaluated in order to observe a hormetic dose response as in toxicology studies (**Figure 1**). The developments of analysis of the hormetic dose responses in plants need rigorous criteria in homogeneous selection of the individuals, several doses of the biostimulant or biological control compound, exposure time, and greater sample sizes (Calabrese and Blain, 2005; Mattson, 2008). Other phenomena such as pre- and post-conditioning must be taken into account when evaluating the hormesis response.

## Plant Recognition and Defense Mechanisms

Plants, due to the lack of mobility, are exposed to adverse environmental factors that cannot be avoided, and having a direct influence on their development. Adverse factors include not only physical conditions such as temperature salinity, drought, radiation, but also plant–organism interaction. Although it is unknown whether the physiological benefits of pathogen challenges fulfill the characteristic pattern of hormesis, the finding that life-history traits can be improved by a single dose of pathogen challenge suggests that immunization as a type of hormesis, can be induced by host responses to pathogen challenge, and even when the source of stress response is not a pathogen, appears to be driven by the expression of genes associated with immunity (McClure et al., 2014). An interesting study in *Drosophila melanogaster* with a single topical dose of dead spores of the entomopathogenic fungus, *Metarhizium robertsii* displayed a close relationship between the defense system and hormesis, suggesting that hormetic responses to stress might be greater in animals lacking functional immune responses and that hormesis should increase susceptibility to infection (McClure et al., 2014).

It is likely that there is a close relation between physical and chemical induced hormesis in plants and plant defense pathways. It has been proposed that low concentrations of toxic metals induce hormetic effects through activating plant stress defense mechanisms (Poschenrieder et al., 2013). Organisms possess genetic information to produce changes in phenotype, which lead the process of moderate adaptation. These plant changes include a range of more effective plant defenses. On the other hand, there are factors that also limit their development, including coexistence with other living organisms, both beneficial and non-beneficial. An important process in the plant–organism interaction is the recognition between self and non-self (Coers, 2013). It is therefore important, after the recognition of the microorganism, to give a response with a high degree of specificity, according to determinate microorganism, depending on one or few genes presented by both plant and pathogen (Brodeur, 2012). Specificity also occurs in the organs, some organisms develop on a single tissue or, in aerial or parts under the ground (Jones and Dangl, 2006; Strugala et al., 2015). The onset of this process is given by physical and chemical signals followed by recognition of MAMPS or PAMPs, and/or DAMPs by transmembrane pattern recognition receptors (PRRs), resulting in PAMP-triggered immunity (PTI) (Sanabria et al., 2010). After this, pathogens release effectors, which are recognized by host causing an effector-triggered susceptibility (ETS), causing disease resistance and HR (Wiesel et al., 2014). Pathogens try to avoid effector-triggered immunity (ETI) through a constant struggle to evade the defense system of the plant by the synthesis of compounds named effectors. This recognition process prevents the spread of disease throughout the plant by restricting the invasion through systemic induced resistance (SIR) (Stotz et al., 2014). The time of inducing the stress responses is important for the plant surveillance, the faster the plant responds to the pathogen attack, the easier is to cope the infection (Newman et al., 2013). After a plant pathogen encounter with its host, host susceptibility decreases to subsequent pathogen attacks. In addition to the foregoing, one of the SIR is systemic acquired resistance (SAR) characterized by giving a perdurable resistance for a long time, characterized by localized necrosis, expression of pathogenesis-related (PR) genes, and accumulation of salicylic acid (SA; Dempsey and Klessig, 2012). The event that results in the encounter between the host and microorganism can provoke symbiosis, disease, or disease resistance; however, non-pathogenic microorganisms can also induce a systemic resistance in plants although to a lesser degree (Pieterse et al., 2014). Products resulting of defense mechanism are cell wall reinforcement, production of ROS, and the synthesis of phytoalexins, and PR protein.

Plant resistance to a pathogenic microorganism depends on their specificity. Differences in the degree in which plants are being infected and failure in infection depend on changes of genotypes caused by evolution in both plants and pathogens (Antonovics et al., 2013). There are two kinds of resistance in plants, host and non-host resistance (NHR), in which responses during infection are very similar (Thakur and Sohal, 2013). NHR is present in entire plant species to a non-adapted pathogen. Two kinds of these have been proposed Type I and II NHR, the first does not produce visible symptoms whereas Type II NHR a rapid hypersensitive response is observed followed by cell death (Cheng et al., 2012). Host resistance, on the other hand, is given by the specificity of the pathogen race or plant cultivar, and is mediated by the interactions of resistance genes I and avirulence genes (Avr), this process is explained by gene for gene model (Cheng et al., 2012). Resistance of the plant and avirulence of the pathogen are present when recognition of the R genes and corresponding Avr genes occurs (Thakur and Sohal, 2013).

## Priming of Plant Defenses

Although plants do not spend in the implementation of defense when there is absence of enemies, when these are presented, plants can suffer irreparable damages during the time required to mount defenses once attack occurs (Frost et al., 2008). As part of evolution, plants have developed a priming process to ward off these dangerous situations. Stress factors have the ability to induce priming including some elicitors derived of organisms such as plants or microorganisms. For example, primed tomato plants with elicitors such as chitosan (CHT), SA, and jasmonic acid (JA) have the ability to promote resistance in plants against a higher stress provoked by *Ralstonia solanacearum*, reducing vascular browning and wilting symptoms of tomato (Mandal et al., 2013).

The process of priming exhibits certain characteristics, it establishes in the exposed tissue to the elicitor and the distal parts, and the response due to the priming defeats a wide spectrum of microorganisms, in posterior attacks the activation is faster, stronger, and last longer or has attenuated repression (Conrath et al., 2015; Martinez-Medina et al., 2016). Pathogen attack on AMF-inoculated in tomatoes provoked strong defense responses by induction of PR proteins, PR1, PR2, and PR3, as well as defense-related genes LOX, AOC, and PAL, in addition, the induction defense responses in AMF pre-inoculated plants was much higher and more rapid than in un-inoculated plants (Song et al., 2015). The main advantage offered by priming is the reduction of the metabolic cost for plants, for example, to attract natural enemies of the herbivore can produce a minor cost than induction of direct defenses and on the other hand the ability to maintain fitness in complex environments (Conrath et al., 2015; Martinez-Medina et al., 2016).

**FIGURE 2:**
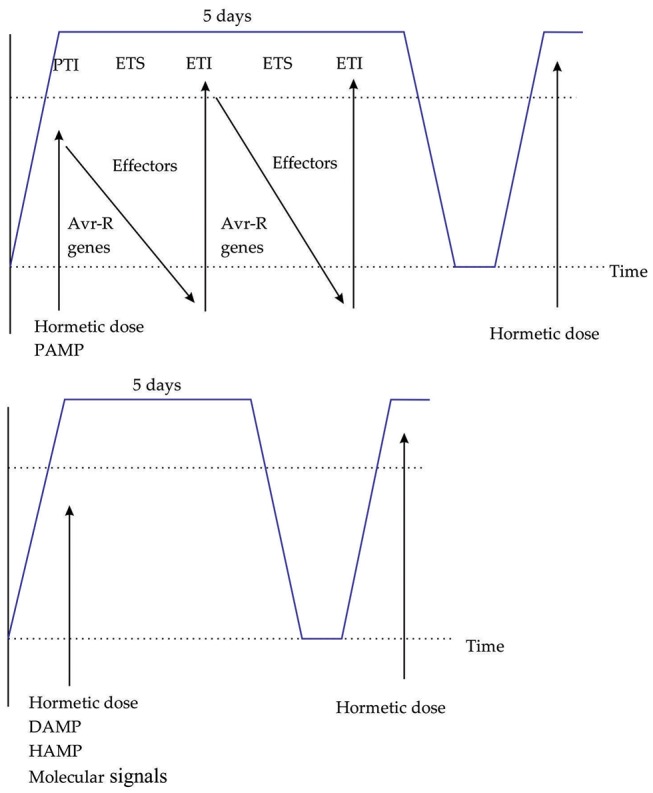
The plant immune system triggered by PAMPS, DAMPS, HAMPS, and molecular signals. **(A)** Zig-zag model describing how plants detect PAMPs dose and induce PAMP-triggered immunity. **(B)** Hormetic dose of DAMP, HAMP, and molecular signals induces a defense response, subsequent responses are greater. Blue line indicates the defense response duration in days.

After experiencing for the first time the stress plants have the ability to respond differently to the following stress exposures (Avramova, 2015). When plants are primed by different stress factors various types of systemic plant immunity can be induced, including SAR and ISR (Aranega-Bou et al., 2014; Pieterse et al., 2014), the above is presented in **Figure 2**. The responsible mechanism for priming in plants has not been completely deciphered. Some of the components playing a central role in SA-mediating priming in *A. thaliana* are the mitogen-activated protein kinases 3 and 6 (Beckers et al., 2009), transcription factors MYC2 (Pozo et al., 2008), elevated levels of PRRs such as FLS2, CERK1, and epigenetic modifications (Jaskiewicz et al., 2011; Tateda et al., 2014). Another advantage for agricultural application is that the priming characteristic in plants can pass down generations, that means an epigenetic component of transgenerational defense priming exist, showing progeny enhanced defense responses (Avramova, 2015). Changes in chromatin structure in responses to environmental stresses are inherited through mitotic and meiotic divisions (Avramova, 2015). Some chemicals have the ability of boosting defense responses and therefore also priming processes, however, the use of chemicals presents negative impacts to environment and also a determined degree of toxicity for plants (Belz and Duke, 2014). Process of priming in plants by the use of elicitors is related to conditioning a term proposed by Calabrese et al. (2007), defined as the process describing when an organism is first exposed to low doses of a stress factor, it has the ability to activate or up-regulate existing cellular and molecular pathways that allows it to withstand subsequent stresses that are more severe.

## Biostimulants and Biological Control Compounds As Factors Inducing Hormesis Responses

Natural compounds called elicitors induce similar defense responses in plants as induced by the pathogen infection. The chemical structure of biostimulants and biological control compounds varies and includes organic molecules for example carbohydrate polymers, lipids, glycopeptides and glycoproteins, or chemical such as SA, CHT, and hydrogen peroxide, among others (Thakur and Sohal, 2013). Several reports have documented the effect of elicitors of biotic origin in plant defense, increasing the levels of H_2_O_2_ in plants (Sharma et al., 2012), turning on the expression of *pal* and *cat1*, indicators of oxidative stress-specific signaling and *pr1* as indicative of biotic stress (Mejía-Teniente et al., 2013), inducing structural barriers, toxic chemicals, and attraction of natural enemies. Evaluation of the effect of elicitors has been limited to the use of phytotoxins. However, some elicitors may have the ability to induce a similar hormetic response in plants, **Table 1**.

**Table 1:** Reports of hormetic and hormetic-like curves under the effect of elicitors of biotic origin..

Treatment/doses	Species/endpoint	Maximum effect	Reference
H_2_O_2_ (0, 0.1,0.5, 1.0, or 1.5 mM)	*Vigna unguiculata* (leaf area, shoot length, root length, shoot fresh weight, shoot dry weight, root fresh weight, and root dry weight)	0.5 mM for all parameters	Hasan et al., 2016
SA (0, 50, 100, 150, 200 mg l^-1^)	Rice seed yield	100 mg l^-1^	Tavares et al., 2014
SA (0, 1.38, 13.8, 69.09, and 138.12 mg l^-1^)	Young barley seedling (length and fresh weight)	SA 1.38 mg l^-1^	Salitxay et al., 2016
JA (0, 50, 100, and 150 mM)	*Calendula officinalis* (cell weight)	50 mM	Wiktorowska et al., 2010
Chitosan (0, 25, 50, 75, and 100 ppm)	*Vigna radiata* (L.) Wilzek dry mass per plant, harvest index, and photosynthesis	75 ppm	Mondal et al., 2013
Chitosaccharides (1, 10, 50, 100, 500, and 1000 mg l ^-1^)	Symbiotic interaction between *Bradyrhizobium* and soybean (number and dry mass of nodules of roots)	100 mg l^-1^	Costales et al., 2015
Fungal elicitor *Verticillium dahilae* Kleb. (0.1, 0.2, 0.3, 0.4, 0.5, 0.6, and 0.7 mg l^-1^)	*Artemisia annua* (cell growth)	0.4 mg l^-1^	Hong et al., 2000
Extract of the polysaccharide fraction of *T. atroviride* D16 (30, 60, and 180 mg l^-1^)	*Salvia miltiorrhiza* (growth of hairy roots at 6, 12, and 18 days)	30 mg l^-1^ (6, 12 days)69 mg l^-1^ (18 days)	Ming et al., 2013
Microbial metabolic products from microorganisms as *Streptomyces* and *Bacillus* (0, 1, 2, and 3 ml l^-1^)	Young barley seedlings (length and fresh weight)	1 ml l^-1^	Salitxay et al., 2016
MeJA (0, 30, 100, 300, and 1000 ml l^-1^)	Two lily genotypes *L. longiflorum*, and *L. speciosum* regenerated *in vitro* (bulblet FW, regenerated bulblets)	30 ml l^-1^ (bulblet FW) 300 ml l^-1^ (bubbles per explant in *L. longiflorum*)	Jasik and de Klerk, 2006
MeJA (1, 5, 10, and 20 ml l^-1^)	Young barley seedlings length and fresh weight	1 ml l^-1^	Salitxay et al., 2016
Pectin (2, 4, and 6 mg l^-1^)	*C. officinalis* suspension cultures (cell growth)	2 mg l^-1^ (12 years 24 h)	Wiktorowska et al., 2010
		4 mg l^-1^ (72 years 96 h)	
Parthenin (12 concentrations in the range of 0.03–6 mmol)	*L. sativa* var. capitata cv. Maikönig or cv. Hilro (root length under different parameters)	0.23–0.65 mmol	Belz and Cedergreen, 2010

### Proposal of Homolog DNA As Elicitor in Plants

DNA is an essential molecule for organisms, which possess the information for survival. As a consequence of evolution, cells are able to detect several pathogen-derived or host, derived substances released when there is damage, including DNA (Hornung and Latz, 2010; Pisetsky, 2012). DNA can act as DAMP, alerting the presence of serf-damage or as MAMP or PAMP if there is the presence of a foreign organism (Gallucci and Maffei, 2017). Recently, studies have demonstrated the inhibition effect of extracellular random fragmented homolog DNA in plants in a dose-dependent manner in comparison with the heterologous DNA (Mazzoleni et al., 2014, 2015). This effect can be biologically general because it occurs in various organisms (Cartenì et al., 2016). DNA recognition by the organism is necessary for the aforementioned process to exist. Recognition of DNA both own and foreign is the task of the PRRs (Gallucci and Maffei, 2017). In mammals, it is known that the recognition is given by Toll-like receptor (TLR9) and cyclic GMP–AMP synthase (cGAS) and absent in melanoma 2 (AIM2), depending on the localization in either the endosomal compartment or in the cytoplasm (Gallucci and Maffei, 2017). It is not clear the mechanism of DNA recognizement by plant cells or the function of extracellular self-DNA in the organisms (Mazzoleni et al., 2014, 2015). Although plants have putative plant PRRs, no extracellular DNA receptor has been identified, but PR proteins are proposed being good candidates as receptors (Gallucci and Maffei, 2017). Some authors consider that the recognition of DNA in plants is similar to that presented by animals through sensors, including TLR9 (Paludan and Bowie, 2013; Pradhan et al., 2015).

Currently there is no information about beneficial effects at low doses in plants. Studies, just reported that low doses of the homologous DNA of an organism has greater damage than the DNA of other organisms (Paludan and Bowie, 2013; Pradhan et al., 2015). It was proposed that extracellular DNA plays a function as DAMP, to certain low doses of the compound. Probably, DNA excreted by plants and further metabolized to sequences of 50–2000 bp have a very specific signature for each species to be recognized as proper by the plant. Bacteria through DNA restriction–modification (R–M) distinguish the same from the strange through DNA methylation. The same effect is observed by TLR9 that specifically recognizes unmethylated CpGs (Krieg et al., 1995; Pohar et al., 2015; Gallucci and Maffei, 2017); while in plants specific responses depend on DNA fragmentation (Gallucci and Maffei, 2017). Thus, DNA methylation patterns could be one possible mechanism for self-DNA recognition in plants, although more research should be addressed in this sense.

This discovery opens new opportunities by exploiting the best characteristics of self-DNA in both agricultural and pharmacological industries, as highly species-specific inhibitory products, limiting the effect for other species (Mazzoleni et al., 2014, 2015). As the next step it is necessary to prove if homolog-DNA possesses a stimulation effect in at low doses. On the other hand, it is also important to elucidate the mode of action of the self-DNA to classify it as a biostimulant, biological control compound, or both. One of the main advantages is the use of self-DNA as elicitors in plants, by inducing machinery of defense and, as a result, all the plant by-products.

## Controlled Elicitation in Plants and Xenohormesis

Nowadays conventional farming practices are aimed to increase yields and decrease losses provoked by pests, diseases, weeds, and workability (Pradhan et al., 2015). To achieve this, the strategy has been reducing stress in crops that in consequence increase the production of primary metabolites thus obtaining yield gain. However, the secondary metabolites in those crops tend to decrease in the edible part (García-Mier et al., 2013). Xenohormesis hypothesizes explain how organisms have evolved to respond to stress signaling molecules produced by other species in their environment (Lamming et al., 2004). Many of the polyphenols are synthesized by plants during times of stress and induce survival and stress resistance of heterotrophs, to this interspecies communication of stress signals is called “xenohormesis” (Howitz et al., 2003). Some of the secondary metabolites as quercetin or resveratrol possess low degree of toxicity, suggesting that the health benefits are not related to mild cellular damage but from the evolution adaptative modulation of enzymes and receptors of stress-response pathways (Howitz and Sinclair, 2008). Resveratrol found in diverse species but it is mainly found in grapes (*Vitis vinifera*), a polyphenol possessing several biological activities, prevent early mortality and help in general health in mammalians (Smoliga et al., 2011).

In contrast, in organic agriculture, as it is done under conditions of constant stress during the development stage, the crops produce a greater amount of nutraceuticals. Several recent works involving the use of biotechnological techniques to establish the efficient production of nutraceutical compounds have been reported, one promising strategy is the use of stress conditions that turn on the defense responses and produce the synthesis and accumulation of bioactive compounds (Naznin et al., 2014; Shrivastava et al., 2015). Resveratrol can be induced in grapes by both biotic such as UV–C radiation and AlCl_3_, or abiotic factors, including fungi, JA, SA, and H_2_O_2_ (Hasan and Bae, 2017). There are various components throughout the process of plant defense, including space-temporal level, post-transcriptional and post-translational modifications, compartmentalization, metabolite stability, substrate availability, among others (Sewelam et al., 2016). On the other hand, diverse aspects also must be taken into account related to the biostimulants and biological control compounds use, such as dose, period of application, specification of its duration, plant age, and developmental stage. It seems that the production of different metabolomics profiles will vary depending on stress circumstances. In this context, the determination of the limits of hormesis is a strategy that can be managed for the generation of agricultural practices that allow to take advantage of the crops, managing an adequate balance of yield and nutraceutical production. Thus, there is a possibility of obtaining health-related products that may be of interest by using different type of biotic elicitors, and then considerable efforts may be focused on the search for new ways to turn on the plant defense and within this context. A search for new sources of biostimulants and biological control compounds will provide the basis to develop a strategy based on inducing plant defense in a controlled manner by understanding the interaction of signal transduction pathways induced by a specific biotic elicitor. This may lead to the synthesis and accumulation of a desired nutraceutical compound and optimize its yield production. In order to increase cultivars production some strategies are proposed:

(1) Avoiding the activation of the defense metabolism until harvest, in order to avoid the defensive reaction of the plant during its development.
(2) Focusing the defensive metabolism effort only in the edible part of the plant and thereby reducing the production of secondary metabolites in non-edible parts.
(3) Differential induction of specific metabolic pathways for the synthesis of nutraceuticals testing with different doses and types of elicitors.
(4) Management of increased elicitors’ doses throughout the phenological stages during plant cultivation. Low doses during vegetative stage, and high doses during fruit development.

Although there has been progress in the characterization of hormetic curves with the application of abiotic stressors, there is still an area of opportunity related to study of biotic elicitors. The above would confer advantages to the strategy presented here because it should influence the absence of negative effects on the environment and, consequently, on health. Some authors have previously recognized the use of biotic elicitors such as a tool to implement sustainable agriculture (Stenberg et al., 2015). The use of biostimulants and biological control compounds for priming activation in agriculture produces a greater increase of yields when they are applied in combination with chemicals than when applied alone (Conrath et al., 2015).

## Conclusion

Based on the abovementioned, there is evidence to suggest that biostimulants and biological control compounds of biotic origin (elicitors) can induce the phenomena of hormesis in plants. Hormesis management by mild stress (eustress) might be a powerful tool in improvement of food nutraceutical quality in crops. In this context, adaptive responses induced by cross talking of stress signals between species (plants and mammalians) can be a powerful tool in improvement of human health.

## Author Contributions

MV-H was responsible for reviewing of literature and writing the paper. IT-P was responsible for the work team and the conception of the research topic. RG-G the conception of the research topic and reviewing of the literature. IM-B, LA-A, RO-V, ER-G, and SR-G were in charge of reviewing the writing.

## Conflict of Interest Statement

The authors declare that the research was conducted in the absence of any commercial or financial relationships that could be construed as a potential conflict of interest.
